# Pan-genome-scale metabolic modeling of *Bacillus subtilis* reveals functionally distinct groups

**DOI:** 10.1128/msystems.00923-24

**Published:** 2024-10-04

**Authors:** Maxwell Neal, William Brakewood, Michael Betenbaugh, Karsten Zengler

**Affiliations:** 1 Department of Bioengineering, University of California, San Diego, California, USA; 2 Department of Chemical and Biomolecular Engineering, Johns Hopkins University, Baltimore, Maryland, USA; 3 Department of Pediatrics, University of California, San Diego, California, USA; 4 Center for Microbiome Innovation, University of California, San Diego, California, USA; 5 Program in Materials Science and Engineering, University of California, San Diego, La Jolla, California, USA; Katholieke Universiteit Leuven, Leuven, Belgium

**Keywords:** *Bacillus subtilis*, metabolic modeling, pan-genome, metabolism

## Abstract

*Bacillus subtilis* is an important industrial and environmental microorganism known to occupy many niches and produce many compounds of interest. Although it is one of the best-studied organisms, much of this focus including the reconstruction of genome-scale metabolic models has been placed on a few key laboratory strains. Here, we substantially expand these prior models to pan-genome-scale, representing 481 genomes of *B. subtilis* with 2,315 orthologous gene clusters, 1,874 metabolites, and 2,239 reactions. Furthermore, we incorporate data from carbon utilization experiments for eight strains to refine and validate its metabolic predictions. This comprehensive pan-genome model enables the assessment of strain-to-strain differences related to nutrient utilization, fermentation outputs, robustness, and other metabolic aspects. Using the model and phenotypic predictions, we divide *B. subtilis* strains into five groups with distinct patterns of behavior that correlate across these features. The pan-genome model offers deep insights into *B. subtilis’* metabolism as it varies across environments and provides an understanding as to how different strains have adapted to dynamic habitats.

**IMPORTANCE:**

As the volume of genomic data and computational power have increased, so has the number of genome-scale metabolic models. These models encapsulate the totality of metabolic functions for a given organism. *Bacillus subtilis* strain 168 is one of the first bacteria for which a metabolic network was reconstructed. Since then, several updated reconstructions have been generated for this model microorganism. Here, we expand the metabolic model for a single strain into a pan-genome-scale model, which consists of individual models for 481 *B. subtilis* strains. By evaluating differences between these strains, we identified five distinct groups of strains, allowing for the rapid classification of any particular strain. Furthermore, this classification into five groups aids the rapid identification of suitable strains for any application.

## INTRODUCTION

*Bacillus subtilis* is the best studied Gram-positive bacterium, putting it in a similar position to the Gram-negative model organism *Escherichia coli* (1). Being originally isolated in 1872, *B. subtilis* was one of the first bacteria studied in detail. The genome of strain 168 was one of the first whole genomes sequenced in 1997 (2). Due in part to its ability to utilize inexpensive carbon sources, such as starch and soybean peptides and its widely adaptable secretion systems (3), *B. subtilis* has emerged as a prominent cell factory for a wide range of industrial applications, including the production of multiple B vitamins, hyaluronic acid, N-acetyl-glucosamine, and polysaccharide degrading enzymes (4). In 2004, *B. subtilis* produced enzymes accounting for over 50% of the market capitalization of industrial enzymes (5). Moreover, as several strains of *B. subtilis* have been labeled “generally recognized as safe” (GRAS) by the Food and Drug Administration (6) and given a “qualified presumption of safety” label by the European Food Safety Authority (7), the bacterium has been widely used as a probiotic supplement for both humans (8) and animals (9).

Moreover, *B. subtilis* produces a number of antimicrobial compounds (10), which have been shown to protect against pathogens, such as *Pseudomonas syringae* in *Arabidopsis* roots (11), *Rhizoctonia cerealis* in wheat (12), *Streptomyces* in potatoes (13), and bulb rot disease in *Fritillaria* lilies (14). Thus, its array of antimicrobial compounds and safety in mammals has garnered its interest as a prophylactic antibiotic alternative to reduce the need for antibiotics in animal industries and thereby slow the spread of antibiotic resistance (15). The potential use of *Bacillus* in the human built environment to control the spread of pathogens is also an area of active research (16).

The metabolism of *B. subtilis* — what substrates it may grow on, and what products it may synthesize under different conditions — is therefore of significant interest to both basic sciences and industry. To this end, genome-scale metabolic models (GEMs) have been reconstructed and deployed to analyze and predict *B. subtilis* metabolism. GEMs are composed of a list of reactions associated with the enzymes and transporters found in a given organism’s genome, connected into a comprehensive metabolic network (17). When provided with constraints on reaction rates to reflect the reversibility of reactions and exchange reactions to represent the availability of nutrients in the media, the models predict the fluxes through each reaction that optimize the growth of the organism. GEMs integrate -omics data and known growth phenotypes to refine and validate the network, enabling them to contextualize data and make further predictions (18).

The first GEM for *B. subtilis*, *i*YO844 (19), one of the first GEMs reconstructed for any organism, has been expanded and refined over time. *i*BSU1103 (20) increased the number of reactions from 1,250 to 1,437 and corrected the reversibility of hundreds of others. *i*BSU1209 (21) further increased the scope to 1,948 reactions. These models have been used to inform metabolic engineering strategies for the production of menaquinone-7 (21), asparaginase enzyme (22), and riboflavin (23). These *B. subtilis* GEMs have mainly centered around the strain *B. subtilis* 168; however, *B. subtilis* strains have been known to exhibit high genomic diversity (24). Thus, secretion of antimicrobial compounds (25) and secondary metabolites (26) can vary significantly between strains.

To investigate the role of *B. subtilis* strains in producing valuable compounds, in interacting with its hosts or microbial communities, and in fighting pathogens, it is therefore important to consider the multitude of strains. Thus, in this work, we expanded the previous GEMs into a pan-genome-scale metabolic model representing 481 strains, thereby capturing the metabolic diversity and secretion capabilities of *B. subtilis*. Similar pan-genome-scale metabolic models have been previously deployed to analyze strain-level differences and patterns of metabolic capabilities in *E. coli* (27), *Salmonella* (28), *Staphylococcus aureus* (29), and fungi (30). We subsequently analyzed the predicted metabolic features of each strain of *B. subtilis* to utilize different carbon, nitrogen, and sulfur sources, their potential to produce four key antimicrobial compounds, and used unsupervised machine learning techniques to divide the strains into five distinct groups.

## RESULTS

### The diversity of *Bacillus subtilis*’ pan-genome and pan-reactome

**Fig 1:**
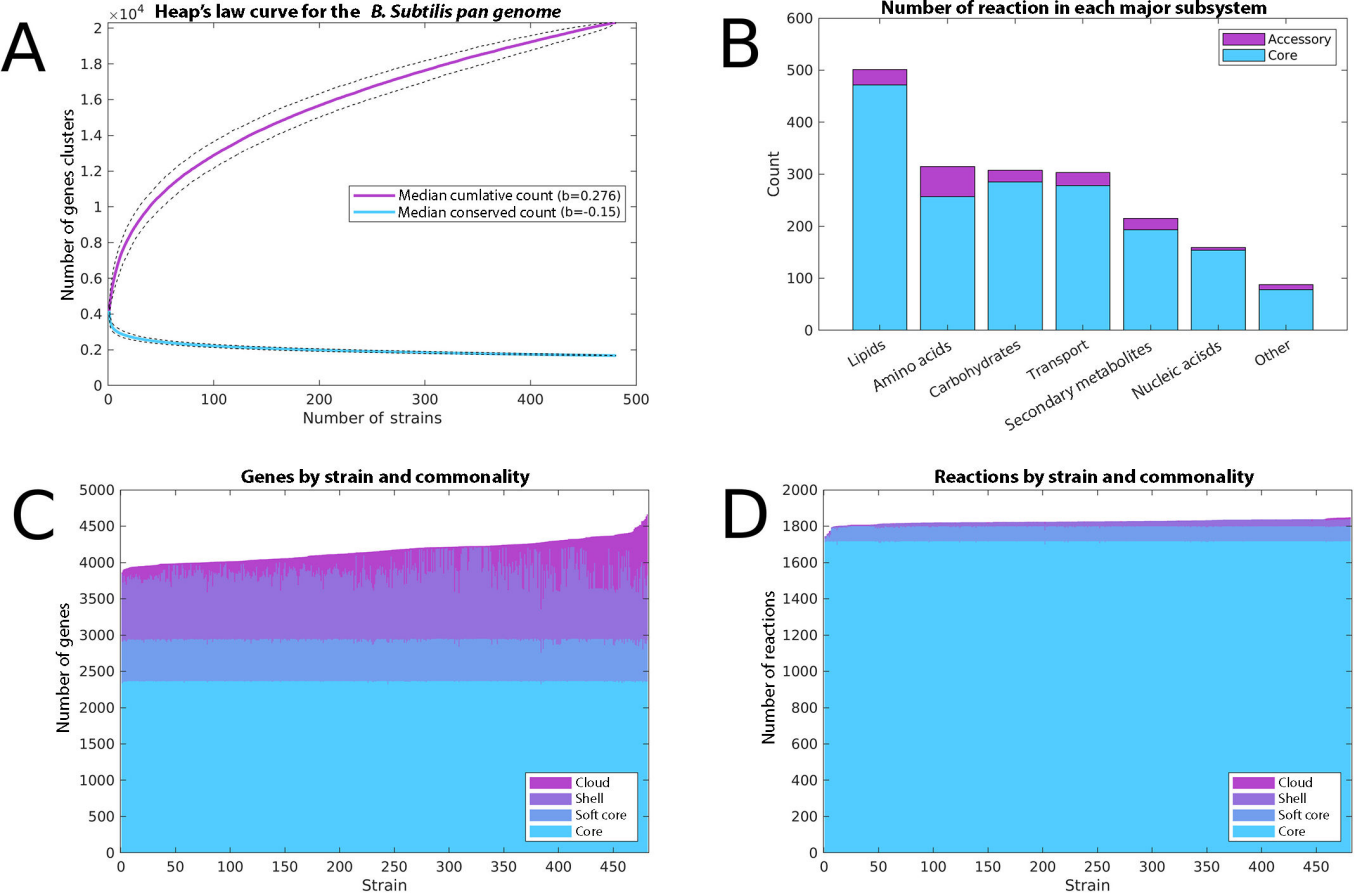
The distribution of the genes and reactions within the pan-genome model across the individual strains. (**A**) Heap’s law analysis of the pan-genome contents. The top curve represents how the number of observed orthologous gene clusters increases with the number of strains. The bottom curve shows how the number of conserved gene clusters (those present in all strains seen so far) decreases. As results vary with the order of the strains, these curves were calculated 1,000 times with randomly permuted strains. The thick, colored lines show the median result, whereas dashed lines represent the 5th and 95th percentiles. The median curves are fit to equations y = ax^b^. (**B**) Each bar shows how many reactions of the pan-model are associated with each major category of the metabolite. The colors then show how they are divided into core and accessory reactions. (**C**) Gene commonality by strain. Each vertical bar represents one strain, ordered by its number of genes. Genes were partitioned by how often they appear in other strains, with the following definitions used in the Roary pipeline (33): Core, 99%–100% of strains; Soft Core, 95%–99%; Shell, 15%–95%; and Cloud, 0%–15%. Note that the genes in any two Core bars will be nearly identical, while those in any two Cloud bars will have little overlap. (**D**) Reaction commonality by strain. The cutoffs and definitions are the same as in (C). Exchange reactions were removed from this analysis.

To construct the pan-genome, we followed the protocol of Norsigian et al. (31), starting with gathering and re-annotating publicly available *B. subtilis* genomes. We then grouped their annotated protein sequences into clusters of orthologous genes to reduce redundancy and identify similar genes across strains (28, 31–33). In total, 481 genomes were incorporated into the pan-genome after filtering for quality (Table S1). The sequence clustering resulted in 20,315 orthologous gene families, reduced from 2*10^6^ individual sequences. These genes can be partitioned into “core” features present in over 99% of strains and “accessory” genes present less frequently. A cutoff of 99% allows for more error tolerance than the frequently used 100%, as erroneous omissions from single genomes will not change the classification (33). Accessory genes are further subdivided into soft core genes (95%–99% of strains), shell genes (15%–95%), and cloud genes (up to 15%) (Fig. 1). We did not enumerate unique genes, as these can be anomalies of sample size. The core genome therefore identifies features shared by almost all strains, representing the unifying features of *B. subtilis* strains. In the *B. subtilis* pan-genome, we identified 2,367 core genes and 17,948 accessory genes (Fig. 1). On average, any two strains share 3,556 genes or 85% of their genetic content. The number of genes observed vs strains is still increasing (Fig. 1A), indicating that the pan-genome is still open and would continue to benefit from additional high-quality genomes.

To reconstruct the pan-genome-scale metabolic model, we followed the protocol of Thiele and Palsson (17), utilizing the representative pan-genome sequences as the input genome. The resulting pan-model, which contains the totality of metabolic reactions across all strains, has 2,315 gene clusters and 1,874 metabolites across the cytosol and extracellular space associated with 2,239 reactions. We also generated a matrix encoding which strains are capable of which reactions, determined by combining the gene–strain and reaction–gene associations (Tables S2 to S4). This matrix is used to transform the central model into one representing any particular strain. When viewed strain by strain, the average strain model has only 64 fewer reactions than the pan-model, at 2,175, an increase of 24% from *i*BSU1209. A total of 92% of this model’s reactions are core features (Table 1), which is notably higher than the 11% core genes (Fig. 1C and D). A randomly selected gene has only a 20% chance of appearing in any given strain, but a gene utilized in the model has a 48% chance of being in any strain’s genome. Furthermore, this is a larger percentage than the 71% of core reactions in the *E. coli* pan-genome (27) and 82% in the *Salmonella* genus reconstruction (28). Among the major pathways, reactions associated with amino acids showed the highest variability between strains, whereas nucleic acids showed the least (Fig. 1B).

**TABLE 1:** Model contents

Feature	Pan-model	Core (99+%)	Accessory (<99%)	Average strain model (standard deviation)
All reactions	2,239	2,067	172	2,175 (12)
Metabolic reactions	1,568	1,386	182	1,514 (11)
Transport reactions	321	273	48	310 (3)
Gene clusters	2,315	697	1618	1,108 (13)
Metabolites	1,847	1,741	106	1,808 (6)

Our model was gap filled to ensure that the individual strains could grow in known growth conditions of *B. subtilis*, including specific defined media (34), prior Biolog experiments for strain 168 (19), and conditions determined by our Biolog experiments for eight additional strains (Experimental model refinement). To fill a strain-specific gap, the most common reactions from the pan-reactome were iteratively added until the model could grow, then trimmed away until a minimal set of new reactions was found (see “Gap filling,” below). Thus, our model presents the most up-to-date reconstruction of *B. subtilis* metabolism, built upon and expanding those that came before it to represent 481 genomes. The final pan-model is available as Table S4 (reactions) and S5 (metabolites) and at GitHub (Data availability).

### Experimental model refinement

For further model refinement, we utilized the Biolog PM1 phenotypic array (35) and determines the capabilities of eight selected strains of *B. subtilis* (i.e. K07, N2-2, 3NA, SMY, PS832, PY79, SU+III, and JH642) to utilize 80 sole carbon sources. These plates, run in triplicate, provided a greatly expanded base of experimentally validated growth or no growth phenotypes at the strain level. The experimental conditions were considered to show growth if the change in OD_700_ was at least 0.05 above the control, and therefore above the detection limit of the plate reader. The corresponding model simulation was checked for a growth rate above 0.01/h to show growth (see “Biolog carbon source experiment and analysis,” below). Adding new pathways and reactions informed by the experiments to correct false predictions refined the model and achieved a predictive accuracy of 92% and a Matthews correlation coefficient of 0.84 across these eight strains. Across 640 data points, the model predicts 398 positive results of which only 17 (4%) are false, in addition to 242 negative predictions of which 32 (13%) are false.

Of the 80 carbon sources tested, only eight showed variability between the strains. These eight metabolites did not represent a particular metabolite class but instead included two amino acids, two carbohydrates, three fermentation products, and one nucleobase. The 32 false positives and 17 false negatives were similarly distributed among varied classes. The full Biolog results are shown in Table S6.

We then curated the model against the carbon, nitrogen, phosphorous, and sulfur Biolog data provided with the original *B. subtilis* strain 168 model *i*YO844, gap filling to improve the accuracy of the strain 168 model and others, achieving a higher prediction accuracy than the prior models (Table 2). We also compared the results of this model to the others in a defined glucose medium with experimentally determined uptake values for strain 168 (34, 36) (Table 2). The minimal glucose medium contains glucose, ammonium, sulfate, phosphate, water, and minerals. Glucose and oxygen were provided at up to 8.7 and 18 mmol/gDW/h to match experimental uptake rates (34, 36). All other components were provided at 100 mmol/gDW/h (Table S4 - see exchange reaction bounds). The growth rate for our model and prior ones were calculated, as well as the variability in the acetate secretion at 95% optimum growth (see “Secretion rates,” below). Our pan-genome derived model for strain 168 achieved more accurate and precise growth and acetate secretion rates in this condition than previously reported models (Table 2), showing an increased model performance in these important model features (growth rates and acid production) on glucose, which is central to carbohydrate metabolism as a whole. Thus, our model presents the most accurate and up-to-date reconstruction of the type strain 168 amongst 480 new strain-specific models.

**TABLE 2:** Growth rates and acetate secretion^*a*^

	Growth rate (h^−1^)	Acetate secretion (mmol/gDW/h)	Biolog accuracy
**Experimental data (34**)	**0.67 ± 0.02**	**4.28 ± 0.29**	**N/A^*b*^**
Average strain model	0.66 ± 0.02	4.34 ± 0.78	N/A
Strain 168 (in this model)	0.67	3.79 ± 0.58	0.75
Strain 168 (*i*YO844)	0.61	4.82 ± 0.96	0.73
Strain 168 (*i*BSU1209)	0.84	0.70 ± 0.70	0.71

^*a*^ The bold indicates one standard deviation.

^*b*^ N/A, not applicable.

### Strains of *B. subtilis* cluster by nutrient utilization profiles

**Fig 2:**
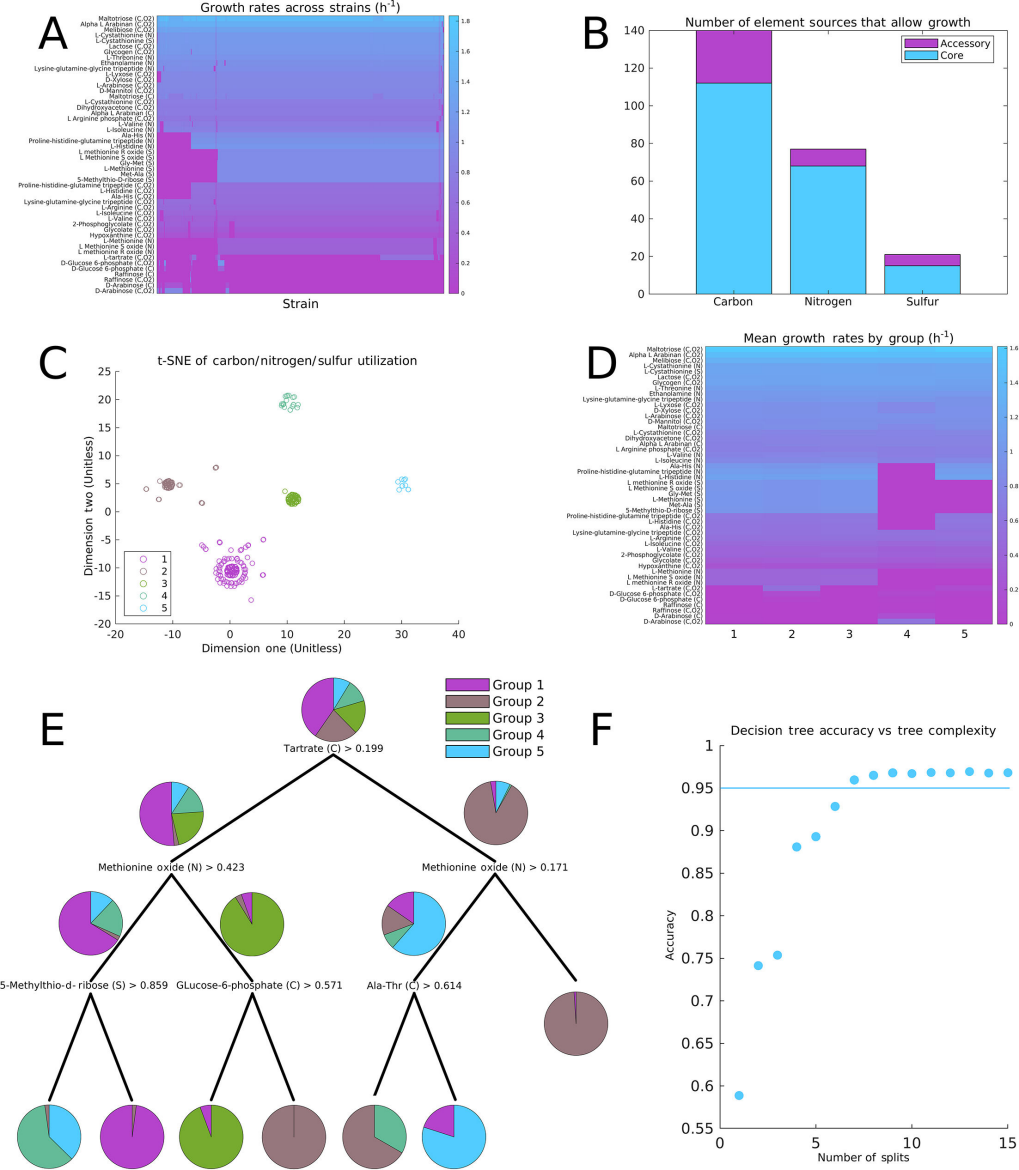
Analyses of the predicted growth rates of the individual strains. (**A**) Predicted growth rates on various carbon sources (with or without oxygen), nitrogen sources (N), and sulfur sources (S). Of all sources in the model, only the 50 with the highest standard deviation of growth rates are shown. The default carbon, nitrogen, and sulfur sources were glucose, NH_4_, and SO_4_; the indicated source replaced only one of these at a time to act as the sole source of the indicated type. The strains and sources have been sorted using agglomerative hierarchical clustering. (**B**) Carbon, nitrogen, and sulfur sources partitioned based on the proportion of strains that could utilize them for growth. (**C**) t-SNE based on the predicted growth rates across all carbon, nitrogen, and sulfur sources. The colored groups were assigned by k-means clustering on the original data. (**D**) The data from A, averaged by the strain groups identified in (C). (**E**) A decision tree trained to predict the strain groups based on growth rate predictions. (**F**) Prediction accuracy of decision trees on a 50:50 train/test split against the complexity of the tree. The accuracy plateaus after six decisions—increasing the tree size past this point would likely lead to overfitting.

Next, we deployed the models to investigate the ability of each strain to grow utilizing every potential sole carbon, nitrogen, and sulfur source (Fig. 2A).

We calculated growth rates on 316 potential carbon sources, 187 nitrogen sources, and 53 sulfur sources. In each condition, the minimal medium was modified by replacing either glucose, ammonium, or sulfate with another nutrient at a maximum uptake of 10 mmol/gDW/h and leaving the rest of the medium unchanged (Fig. 2A). Of these 316, 93 carbon sources could be used by every strain, incuding the monosaccharides glucose and fructose, 19 individual amino acids or short peptides, and malic acid, which *B. subtilis* is known to prefer over other many carbon sources (37). Moreover, 65 nitrogen sources could be used by all strains; in line with previous experimental characterization, these included a wide range of amino acids (38), various nucleobases (39, 40), and ammonium, nitrate, nitrite, and urea (41).

With these results, we categorized nutrients for *B. subtilis* into core and accessory nutrients, defined identically to the core and accessory genes: if at least 99% of the strains grow using a particular nutrient (predicted growth rate >0.01 h^−1^), it is considered a core nutrient; otherwise, it is considered an accessory nutrient (Fig. 2B). These core nutrients, similar to the core genes, therefore identify compounds that an overwhelming majority of *B. subtilis* strains may use, forming a set of unifying and identifying features. The diversity of growth rates is proportionally higher than that of the reactions, whereas ~92% of reactions were in the core reactome, only 80% of carbon sources and 71% of sulfur sources were in the core set of nutrients. The remaining 8% of reactions that comprise the accessory reactome are therefore enriched in likelihood to be impactful in whether a given strain can utilize each nutrient, in contrast to the reactome as a whole. A high proportion of the accessory reactions are involved in transport and the initial steps in metabolizing these diverse compounds and not, for example, in the synthesis of secondary metabolites uninvolved in growth.

Many of the variable carbon sources are di- and tripeptides, with their use being dependent on the presence of specific peptidases. Other carbon sources that showed variability between strains include the carbohydrates arabinose and glucose-6-phosphate and the fermentation products acetate and tartrate. Thus, although many carbon sources form a conserved basis of metabolic possibilities, *B. subtilis* still shows variability in the utilization of simple carbohydrates and bacterial fermentation products. This is indicative of potential differences in how the strains interact with their environments, as different strains may compete with other microbial community members for these carbohydrates or utilize their byproducts differently.

Therefore, we next investigated whether these variations are uncorrelated, or if there are distinct patterns of growth phenotypes within the strains. We first performed dimensionality reduction *via* t-stochastic neighbor embedding (t-SNE) generated from the combined growth rate data for carbon, nitrogen, and sulfur sources (Fig. 2C). This generated five clusters of strains, labeled in order of size (Table S7). When the k-means with five groups was applied to the original growth rate data, the group assignments were the same, and thus the division was not an artifact of t-SNE. The average growth rates in each group show distinct variations, with groups 4 and 5 growing on fewer nutrients overall, groups 1–3 varying in their utilization of glucose-6-phosphate and tartrate, and 41 compounds showing at least 1% variability between groups (Fig. 2D).

Although t-SNE produces clean separation, it lacks interpretability—one cannot extract which features lead to which group assignment. To extract which differences cause these groups to separate, we utilized decision trees. Decision trees divide data based on a flowchart of one property at a time, making them readily interpretable, though at a cost of reduced accuracy. Despite the simplicity of decision trees, a tree with only seven splits yielded a 95% prediction accuracy (Fig. 2E and F). Therefore, the divisions between the strains can be cleanly explained by their growth on a few nutrients. This method also achieved an accuracy score of 80% when only considering binary growth or no growth predictions. The defining metabolites include tartrate as a carbon source, which is utilized by only a few *B. subtilis* strains among all *Bacillus* or genera of any bacteria (42). Another key metabolite is methionine sulfoxide, the utilization of which is associated with motility and redox balance in *Bacillus cereus* (43).

### Secretion potential is related to growth phenotypes

Having defined five groups based on growth rate data, we then examined differences in the end points of metabolism, i.e., which metabolites can be secreted, determined by examining which metabolites could have an efflux into the medium in the model. We simulated each model in minimal glucose medium, then identified the potentially secreted metabolites through flux variability analysis. The growth rate was then fixed to be at least 95% of its maximum to capture optimal and suboptimal network states (44, 45). This value was selected as it allowed for the same set of reactions to be active as when no growth was required, yet still requires growth to be near optimal. Then, to assess which metabolites could be produced and in what amounts, we optimized each strain model with each exchange reaction as the objective (see “Secretion rates,” below). As GEMs generally have a wide range of fluxes possible at or near the optimal growth rate (46), they do not make specific predictions for secretion rates. Thus, these values represent the maximum potential secretion rates. For all strains, the metabolites with the highest secretion rates were water, carbon dioxide, and acetate. The most variable secreted metabolites by standard deviation included ethanol, which is known to be consumed by bacteria in many different environments, including soil and the human digestive system (47–49). Various amino acids followed ethanol as the most variable. Thus, these results help outline how different strains of *B. subtilis* may impact their environment through producing different metabolites in addition to consuming different nutrients.

**Fig 3:**
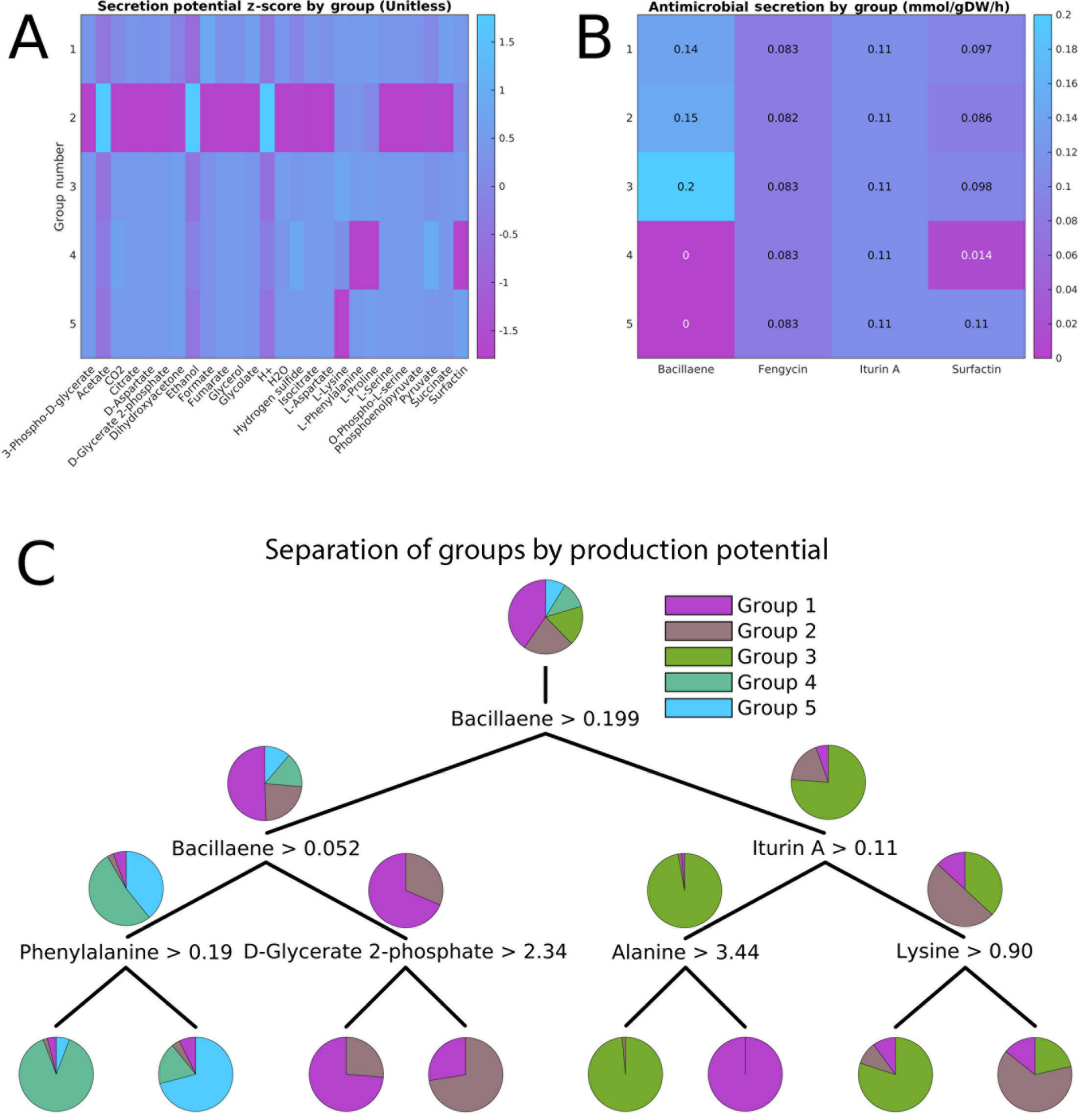
Metabolite secretion predictions. (**A**) Z-scores of maximum secretion rates by group. Only metabolites with a non-zero standard deviation are shown. (**B**) Mean maximum secretion of the antimicrobial compounds in the model by group. (**C**) A decision tree showing how the previously defined groups, although built from growth rates, can be readily predicted by secretion capabilities as well.

We then averaged these secretion rates by the groups defined by the nutrient utilization profiles (Fig. 3A). These groups, defined on growth rates, still visibly separate based on secretion rates. Distinct differences between the groups also emerge with respect to the secretion of the four antimicrobial compounds bacillaene, fengycin, iturin, and surfactin (Fig. 3B). Groups 4 and 5 lack the genes to produce bacillaene at all, whereas group 3 could produce the most bacillaene without sacrificing its growth rate. Other differences are present, but the effect size is smaller compared to these. Thus, the ability to utilize different nutrients is correlated to the ability to produce these antimicrobials. Other differences were observed between the groups among the complete set of potentially secreted compounds, allowing for the groups to be separated with high prediction accuracy (Fig. 3C). In particular, group 2 lacks the pathways necessary to secrete many metabolites the other groups could produce, whereas other groups could be separated by their ability to produce certain amino acids, including alanine, lysine, and phenylalanine.

### Essential pathways and reactions predict growth phenotypes

**Fig 4:**
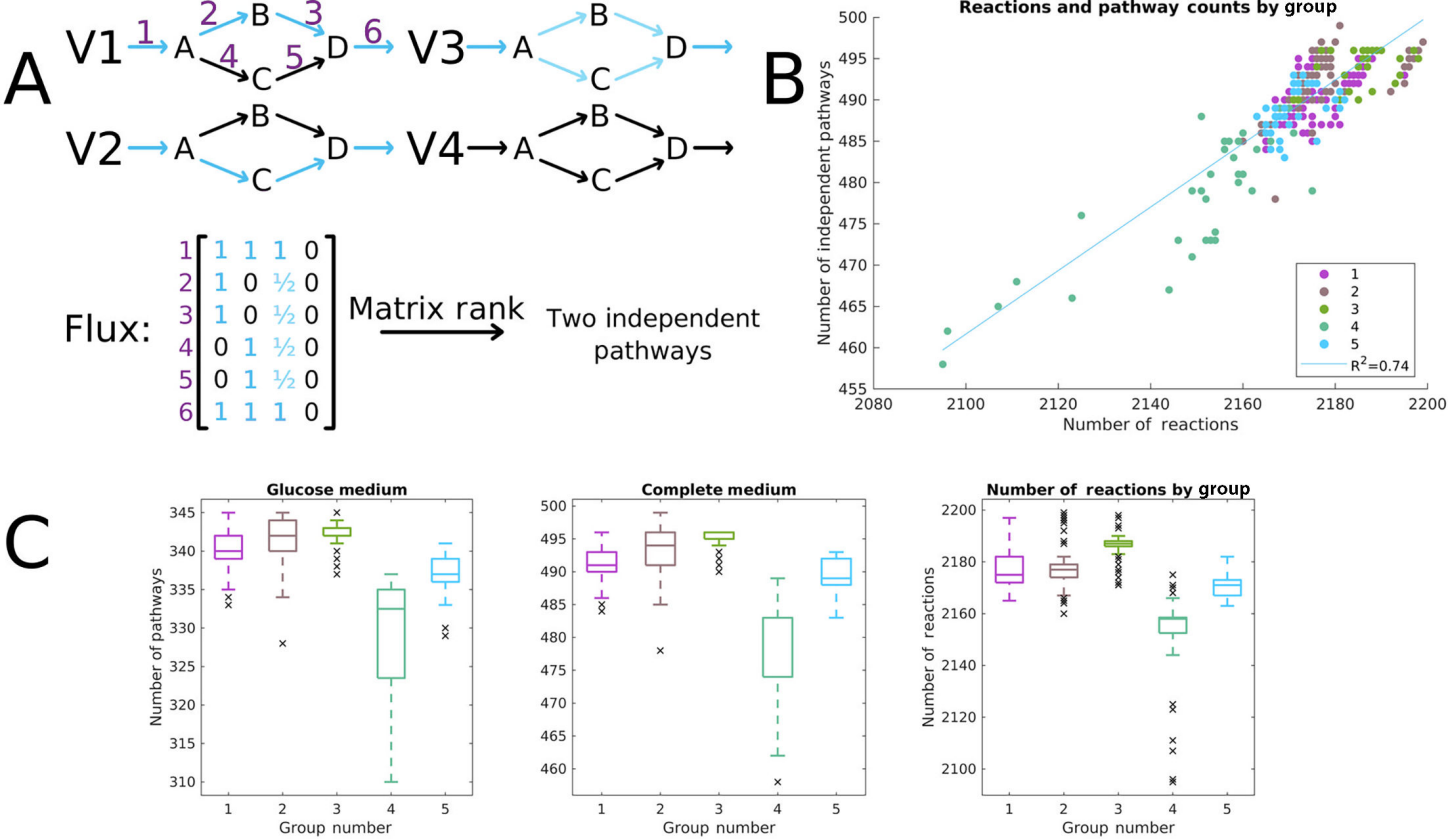
Pathway enumeration statistics by group. (**A**) An example of the calculation of independent pathways. Active reactions are shown in blue. Visually, there are four main network states: metabolite D can be produced from either the upper or lower set of reactions (V_1_ or V_2_), both sets at once (V_3_), or both can be inactive and no D can be produced (V_4_). However, V_3_ and V_4_ can be expressed as combinations of the other two pathways, and therefore are not linearly independent. Thus, there are only two pathways present here per our definition. This can be found easily by calculating the rank of the data matrix of fluxes found in different instances. No matter what linear combinations of these pathways appeared in the flux matrix, and no matter how many samples there were, the rank would always be two. (**B**) A scatter plot of the number of reactions and number of independent pathways, colored by group. Note that group 4 lags behind the others on both axes, whereas groups 2 and 3 are at the higher end. Although the number of reactions and number of pathways are predictive of each other (R^2^ = 0.74), some reactions open multiple pathways and provide no new options to the organism, requiring examination of the full model to calculate this emergent property. (**C**) Boxplots of the number of pathways in each group in the glucose medium and the complete medium (all exchanges open). All except for groups 1 and 2 in the glucose condition are statistically different from each other at a significance level of 0.001.

As shown in Fig. 1C and D, the number of genes and reactions varies significantly between strains. Moreover, some groups of strains utilize fewer nutrients (Fig. 2), suggesting that some strains have fewer functional pathways available to them. To investigate this further, we performed flux sampling on all 481 individual models to assess which had the most potentially active pathways. The term “pathway” typically refers to a semi-arbitrary collection of associated reactions, but here, we use a quantitative definition for “pathway” similar to that of Bordbar et al. (50). Specifically, a pathway is defined as a linearly independent set of reaction rates (Fig. 4A) achievable within the metabolic network. Importantly, we define them to be linearly independent so that potential pathways constructed by merging two or more other pathways or taking an existing pathway in the reverse direction are not counted. The overall number of pathways thus provides a direct measure of the number of metabolic choices the organism has available to it, as well as an indirect measure of its robustness and metabolic adaptability.

Flux sampling yields a large data matrix of possible sets of fluxes, where each row is a sampled metabolic state, and each column is a reaction; this captures the variability in all the reaction rates. Then, the number of linearly independent pathways may be found by calculating the rank of this matrix. We computed these values for all strains in minimal glucose medium and in a complete medium with all modeled extracellular metabolites available in unlimited quantities (see “Pathway enumeration,” below). In each case, the variability in the number of pathways was proportionally higher than that in the number of reactions. For the number of pathways, the coefficients of variation (mean divided by standard deviation) were 0.0143 and 0.0105 for the glucose and complete medium, respectively; for the number of reactions, it is 0.0051. Each variable reaction is on average associated with two or three new pathways, meaning these accessory reactions tend to be positioned at branch points within the metabolic network, with the potential to create multiple new options for the cell to utilize.

Once again, we examined if these pathway-level differences correlate with the five previously defined groups of strains (Fig. 4B and C). These data aligned well with the previously defined groups despite them being built on entirely different data. The number of pathways was significantly different (*P* < 0.001) in both conditions between almost all pairs of groups. Group 4, which showed growth on the fewest nutrients, had the lowest number of pathways. Its median value was below 93% of values from other groups. Similarly, the median of group 3 was higher than 86% of all other data points. These values in combination with the growth data help to characterize the strains of *B. subtilis* as generalists or specialists, with some having many more metabolic capabilities and potentially utilizable nutrients available to them.

The number of essential reactions provides another way of assessing metabolic robustness. We determined gene essentiality through single reaction deletions on every reaction in every strain model in minimal glucose medium. If the growth rate was reduced below 0.01 h^−1^ by the deletion of a reaction, it was labeled as essential for that strain model (Table S8). Of the 186 reactions that were essential for at least one strain, 156 were essential for at least 99% of strains. These reactions define the core set of metabolic transformations that all *B. subtilis* strains are dependent on. When considering reactions that when deleted reduce the growth rate by 1% or more, there are 216 impactful reactions of which 185 are core reactions.

In addition, there are reactions that are consistently essential for strains in specific groups. Reactions S-ribosylhomocysteine lyase (RHCYS), methylenetetrahydrofolate reductase (MTHFR3), and S-adenosylhomocysteine nucleosidase (AHCYSNS ) are each essential for most strains in every group, except group 3. Citrate synthase and pyruvate carboxylase are essential for all members of group 4. The other groups have fewer distinguishable essential reactions with smaller effect sizes, but there are still differences present.

### Diverse phenotypic features predict consistent group assignments

**Fig 5:**
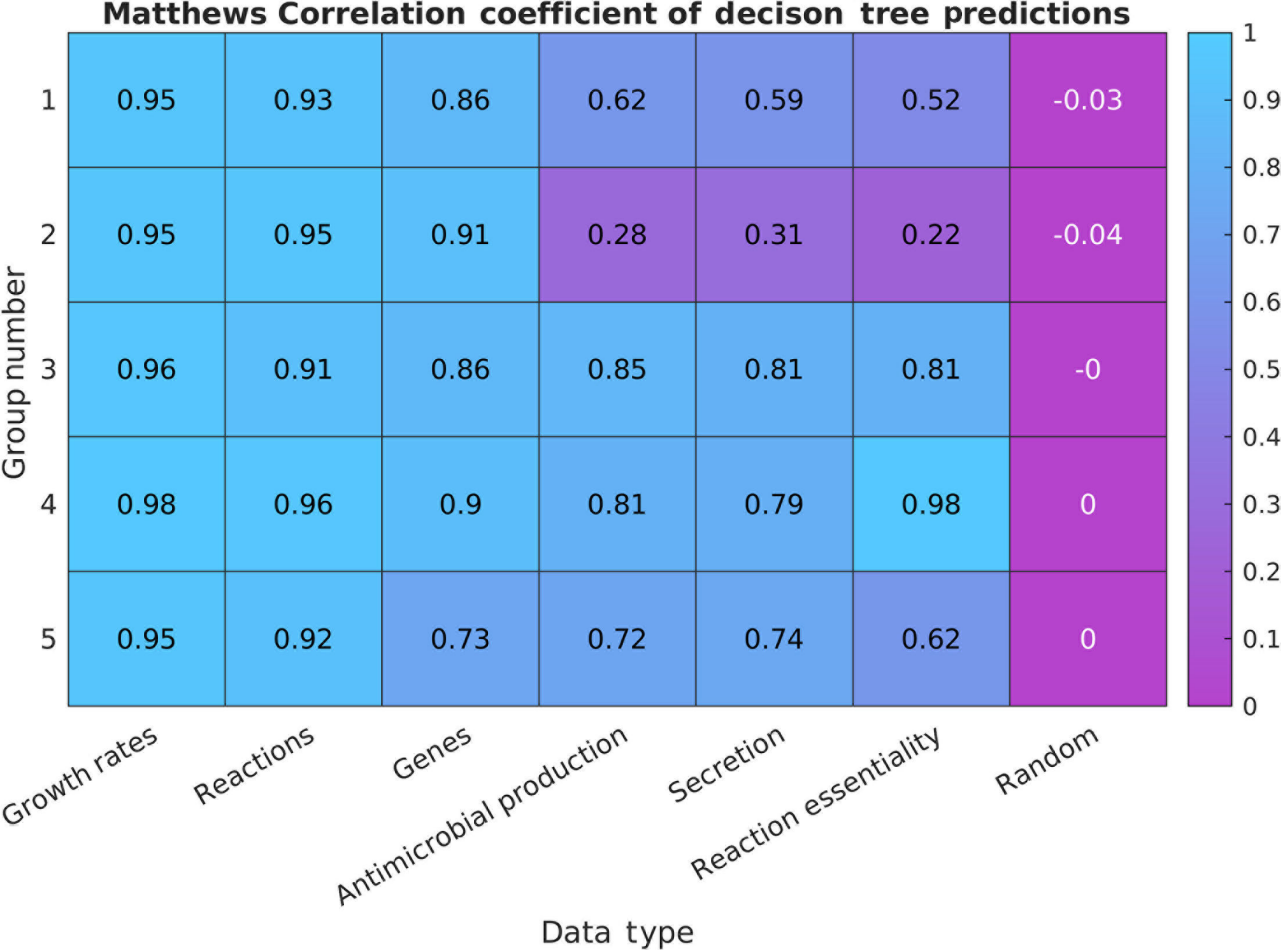
Matthews correlation coefficient (MCC) values from the predictions of each group based on decision trees built from each data type. The data types are sorted by average MCC value. A column built on uniform random data is appended for reference. The values shown are the averages across 100 replicates with randomly assigned 50/50 train/test splits.

Given that data regarding every metabolic feature correlated back to the groups defined by growth rates on different nutrients, we quantified just how predictive these different features are of the group assignment. To measure this, we built decision trees to predict the group assignment based on each of several data types one at a time. After training the tree with a random selection of half the strains, we used the tree to predict the labels on the remaining half. Then, we calculated the number of true and false positives and negatives for each group. These were then combined into the Matthews correlation coefficient (MCC) to represent the overall accuracy of the predictions (Fig. 5). The MCC performs better than other measures of prediction accuracy (such as sensitivity and specificity) when the group sizes are imbalanced (4), which is of relevance here as the largest group is four times larger than the smallest.

As expected, the growth rate predictions performed the best, as the group assignments were built on the growth data. However, the remaining data sources still had significant predictive power. Decision trees built using reaction presence/absence yielded MCC values above 0.90; gene presence information achieved an average of 0.85 across the groups. Examining only the secretion rates allowed for separation of all groups except 2, with an average MCC of 0.65. Surprisingly, focusing only on the secretion of the four antimicrobial compounds reduced the MCC by a few percent. Finally, the reaction essentiality was the most variable, with nearly 100% predictive power for group 4, as its strains had many essential reactions the others did not rely on, but an MCC of only 0.22 for group 2.

Thus, these groups that emerged from predicted growth rates are deeply related with all of these other features. No matter which of these features are considered, similar groupings emerge, and strains that have certain characteristics when viewed from one perspective are highly likely to vary in the same ways when viewed from any other. Thus, we propose to divide these strains into the five groups, characterized by these defining metabolic traits.

## DISCUSSION

Here, we present a comprehensive model of *B. subtilis*, containing new reactions and features when compared with previous models, including the synthesis of four antimicrobial compounds. Furthermore, we extended the model to a pan-genome of 481 strains of *B. subtilis*. We have thus expanded its predictive capabilities beyond the core laboratory strain 168 to yield analysis of strain-to-strain differences in resource usage, growth rates, and secretion. These differences reflect how different strains have adapted to their environments, providing mechanistic insights into the distribution of the ubiquitous *B. subtilis. B. subtilis* strain-level differences reflect their adaptations to environmental differences in nutrient availability and microbial community composition.

We found that *B. subtilis* naturally divides into five groups based on simple rules for which nutrients it can or cannot use. The specific metabolites that divided these groups have been previously identified to be of importance to the genus *Bacillus* (42, 43). Arabinose, utilized efficiently by group 4 and poorly by group 1, has been shown to be impactful on the activity of sporulation pathways (51). Group 1 is distinguished by its ability to utilize 5-methyl-D-ribose for sulfur, whereas group 4 distinctly lacks this ability (Fig. 2E). This metabolite is associated with the starvation response in *B. subtilis* (52). Moreover, it can be produced by *E. coli*, *Clostridium pasteurianum*, and *Saccharomyces cerevisiae* — important members of gut and environmental microbiomes. 5-methyl-D-ribose is also consumed by the soil and human bacterium *Klebsiella pneumoniae* (53); this metabolic difference therefore provides an avenue for differences in response to sulfur starvation in *B. subtilis* strains and their metabolic competition with other prominent microbes. Flux through glucose 6-phosphate has been shown to improve riboflavin production in *B. subtilis* (54), adding relevance to group 2’s high efficiency at utilizing this substrate. Isoleucine usage is variable across groups both as a carbon and nitrogen source, whereas its utilization has been shown to alter fatty acid production and membrane composition in *B. subtilis* (55). Finally, differences between groups in consumption of dietary compounds or bacterial products, such as mannitol and acetate, have implications for that group’s ability to compete and exchange nutrients with the surrounding community.

The five groups defined here consistently align well with other key metabolic features, including which compounds each strain may secrete and which reactions are essential for their growth, allowing us to thoroughly characterize the groups in multiple ways. Group 3 has relatively high hallmarks of robustness, having the fewest essential reactions and many of the highest growth rates assessed; group 4 meanwhile was the least robust by these measures, and group 1 members exhibited reduced growth on many carbohydrates. Strains of group 5 could use the fewest sulfur sources, but produced the most surfactin and propionate by a wide margin. Group 2 strains were catabolic generalists, capable of utilizing the most nutrients. However, strains in this group did not have especially high growth rates and could produce the fewest compounds. This aligns with previous work suggesting that being a generalist in one feature may come with trade-offs limiting breadth in others (56). Additional trade-offs in nutrient utilization, motility, and spore quantity and quality have previously been observed in studies on evolution in *B. subtilis* (57–59).

Thus, the diversity of *B. subtilis* strains can be condensed into features that separate these five groups. These strain differences likely reflect the abundance of strains in microbial communities and thus the models can provide a mechanistic basis to unravel the role of *B. subtilis* in various microbiomes.

## MATERIALS AND METHODS

### Pan-genome generation

The *B. subtilis* pan-genome was generated using the BGCFlow (60) pan-genome software pipeline. This pipeline is a composition of several established genome analysis functions, which streamlines the pan-genome assembly and annotation. The accession numbers for all publicly available genomes from NCBI labeled as *B. subtilis* as of 20 October 2022 were gathered as the input into the pipeline. Within the pipeline, the genomes were given CheckM (61) quality scores. Only “high” and “medium” quality genomes with completeness above 98%, contamination below 3%, and fewer than 100 contigs were kept. This resulted in 481 genomes in the final list (Table S1).

The genomes were then annotated using Prokka (62) to assign functions to the genome sequences. These annotated sequences were then fed into Roary (33) using 95% similarity for clustering. The result of this was the final gene–strain association matrix, identifying which of the final 20,315 gene clusters were found in which of the 481 genomes. Roary also produced a list of 20,315 representative sequences, one for each cluster, which was used in all the further analysis as the pan-genome for BLAST and other purposes (Table S9). The annotation for each cluster representative was chosen by selecting the most frequent annotation among the cluster constituents.

### Metabolic model reconstruction

The metabolic model was made roughly following the workflow provided by Palsson et al*.* (17). Unlike a typical reconstruction, the input genome used here was not that of the individual organisms, but the pan-genome of 20,315 representative sequences. These sequences were compared with the genes used in template models using protein BLAST. The template models used included two prior reconstructions of *B. subtilis*, the core original model *i*YO844 (19), and the more comprehensive *i*BSU1209 (21). Other templates included the high-quality *E. coli* model *i*ML1515 (63), two models of the Gram-positive *S. aureus* [*i*SB619 (64) and *i*YS854 (65)], a model of the Gram-positive *Lactococcus lactis* [*i*NF517 (66)], and other high-quality models from the BiGG database (67). High-quality draft models, such as *i*ML1515, were included despite being Gram-negative to increase the chances of finding all relevant reactions that would not be found in the limited number and scope of manually curated Gram-positive reconstructions. All reactions were later manually curated, removing erroneous reaction–gene associations. In the final reconstruction, only 176 metabolic reactions came solely from Gram-negative templates. The BLAST parameters used were an E-value no greater than 10^−30^ and an identity of no less than 30%. Any reactions for which all components of the GPR had a BLAST match in the pan-genome were collected to form the first draft model. The biomass reaction was taken from *i*YO844. Exchange reactions were added for each extracellular metabolite in the model. Additional reactions were identified by finding pan-genome entries with enzyme commission numbers that were not yet used in the GPR for any reactions. Then, the pan-genome was then compared against entries from the Transport Classification Database (68) using the same BLAST criteria to find additional potential transporters.

These automatically constructed GPRs were then manually curated, with the proposed reaction associations compared with the annotations of the sequences or functions established in the literature. Enzymes with multiple assigned reactions were assumed to be capable of performing them all, unless refuted by literature or specificity of EC number. Reactions with GPRs that apparently did not match were kept when literature supported multifunctionality of the relevant enzymes. Reactions whose GPRs involved proteins with vague names (e.g., “carbohydrate ABC transporter”) were kept but noted as low confidence. Reactions involving the periplasmic space, which the Gram-positive *B. subtilis* does not have, were mapped to the cytoplasm instead. The original model *i*YO844 contained 117 metabolic reactions and transporters with no assigned genes. Using the updated *B. subtilis* models and searching the abundance of biochemical literature published since the original’s creation, we assigned new GPRs to many of them, reducing the number of gaps. Finally, the reaction–strain association matrix was made by identifying which strains had genes that could satisfy each of the GPRs. Strain-specific models are made by removing reactions not associated with the strain of interest from the overall pan-model.

The metabolites in the model were assigned formulas and full names by mapping them over from the template models, with priority given to the *B. subtilis* models’ information. The metabolite formulas were unified to ensure no reactions were unbalanced. Reactions that were unclear or unbalanced by nature (such as R00991 in KEGG) were removed from the model. Reaction bounds were chosen by following what the majority of template models used. Every reversible reaction was given default bounds of ±1,000 mmol/gdwt/h; irreversible reactions were given bounds of [0, 1,000] mmol/gdwt/h. The default glucose uptake bound was set to 8.7, the value calculated experimentally by Tännler et al*.* (34). Other exchanges for minerals, ammonium, and oxygen were left fully open, with bounds of ±1,000. All non-media exchange reactions were given bounds of [0, 1,000] to allow any metabolite to leave the system.

Our model contains reactions representing the secretion of four antimicrobial compounds: surfactin, bacillaene, fengycin, and iturin A. These were selected for being some of the best studied and most important for competing with major pathogens. All but bacillaene are non-ribosomally constructed lipopeptides, whereas bacillaene is a polyketide. The genes and precursors required are known, but the step-by-step pathways for producing these are not well characterized (10). They are therefore represented as lumped reactions from precursors to the final product.

The oxidative phosphorylation reactions had to be modified to prevent infinite ATP generation. The ATP synthase reaction was originally powered by extracellular hydrogens. However, many metabolic reactions and transporters from models without ATP synthase create or transport hydrogen atoms, such as D_LACt2 and MTHFC. These reactions can be used in conjunction with each other to create a cycle that generates arbitrarily many extracellular hydrogen atoms. As individual hydrogen atoms are not usually metabolically relevant, this does not usually cause any problems. However, when the proton-driven ATP synthase is present, this allows for infinite energy generation. To combat this, reactions that typically pump hydrogen for oxidative phosphorylation purposes, such as cytochrome bd oxidase (CYTBD), were modified to instead create a dummy metabolite “oxphosH,”' which may then be consumed by ATP synthase. This way, the oxidative phosphorylation pathway remained functional without the need to edit hundreds of reactions from various sources.

### Biolog carbon source experiment and analysis

Metabolic characterization of eight *B. subtilis* strains obtained from the *Bacillus* Genetic Stock Center (Columbus, OH) was carried out using Biolog PM1 plates obtained from BIOLOG Inc. (Hayward, CA). Each strain was streaked on LB agar plates at 37°C, and colonies were subcultured once on an additional plate before seeding, with Biolog plates being prepared according to the manufacturer’s protocol for Gram-positive bacteria using redox dye F (69). Once inoculated, the plates were incubated at 30°C for 48 h, with absorbance readings being taken at 592 nm to assess metabolic activity and 750 nm to assess cell growth at 0 and 48 h. Absorbance was measured using a Promega GloMax-Multi + microplate reader (Madison, WI). Biolog plates were run in triplicate for each strain, with *B. subtilis* strains K07, N2-2, 3NA, SMY, PS832, PY79, SU+III, and JH642 being evaluated.

Growth was determined by finding the change in OD_750_ between the initial and final time points. The change in OD_750_ for the negative control was subtracted from the change in OD_750_ for each nutrient in the corresponding replicate and strain data. If this value was above 0.05, the detection limit of the machine, in two or three of the triplicates, then that strain was considered to have shown growth on that nutrient. For the corresponding *in silico* analysis, each nutrient was provided at a maximum uptake rate of 10 mmol/gDW/h, replacing the currently active exchange reaction for that type of source (C, N, P, and S). If the model then predicted a growth rate greater than 0.01, the model was considered to show growth on this nutrient.

### Gap filling

After the initial curation, the pan-model could grow. However, only about half of the strain specific models could grow, so gap filling was required to make the metabolic network functional. Most individual strains that could not grow were missing one or two essential reactions. To identify and fill these gaps, an iterative method was used. Core reactions identified as essential for growth in the pan-model were added one at a time until the strain model could grow. These added reactions were then removed one at a time, so that the minimal set of added reactions necessary for growth could be found. By selecting from core reactions essential to the pan-model, we selected reactions with a high likelihood of being present in any given *B. subtilis* strain, increasing the odds we could find genetic support.

We then attempted to find genome support for these reactions. If none could be found, these reactions were rejected, and gap filling was repeated with these reactions excluded. If no genomic support could be found, then the smallest possible set of new reactions was added without annotation. This set was found by adding every reaction from the pan-model, regardless of frequency, then trimming away reactions starting with the least common. These strain-specific gaps are marked in the GPRs as “strain_gap(x)” to note that strain x required this reaction for gap filling.

Finally, we gap filled the selected strain-specific models so that they could reflect the growth or no growth phenotypes found in our Biolog data, so that the strain 168 model would best reflect the Biolog data provided with the *i*YO844 model.

### Growth predictions

By default, the growth medium in thae model is unlimited minerals (Ca, Fe, K, Mg, Na, and Pi), water, carbon dioxide, sulfate, and ammonium. To match experimentally observed uptake rates, oxygen is provided at a rate of up to 18 mmol/gDW/h and glucose at 8.7 mmol/gDW/h. The model file in the supplementary information is set to these values. When assessing if a strain model could use a particular carbon source, the uptake rate for glucose was set to 0. Then, the uptake rate for the source of interest was set to 10 mmol/gDW/h and the model optimized with FBA. (“FBA = optimizeCbModel(model)”). Then, the growth rate was extracted. If it was above 0.01, the model was considered to have shown growth; otherwise, it was labeled as no growth. In this way, all carbon sources tested were sole carbon sources. The process for nitrogen and sulfur proceeded similarly, but with NH_4_ or SO_4_ being set to 0.

### Clustering

The five groups were assigned based on t-SNE and k-means clustering. First, the matrix representing the predicted growth rates of each strain using each nutrient (with carbon, nitrogen, and sulfur data concatenated). A two dimensional t-SNE embedding was then computed in MATLAB (“X = tsne(growth_data)”). The groups were then split b k-means (“group = kmeans(X,5)”), which aligned perfectly with the groups observed in the plot. Even on repeated recalculation, the same five visually distinct groups appeared, so no analysis was done to optimize the number of groups. The group labels were sorted by group size for organization.

### Decision trees

When performing any decision tree analysis, the data and group assignments were first split into two randomly assigned halves for training and testing. First, the tree was built using MATLAB’s built-in function (“mdl = fitctree (training_data, training_labels, ‘MaxNumSplits’, 10)”). Then, this tree was used to predict the group assignments of the remaining data (“predicted_labels = mdl.predict(test_data)”). Prediction ability was measured with the Matthews correlation coefficient for each group. Within each group, the confusion matrix of true and false positives and negatives was computed. The MCC was found by the formula $MCC=\frac{TPxTN-FPxFN}{\sqrt{}}$. All reported MCC values were the average from 100 random train/test splits.

### Secretion rates

We calculated the variability of the secretion rates near the optimal growth rate. To calculate the secretion potential for a given strain, we first optimized the model in its default minimal glucose medium. Then, we forced the growth rate to be within 95% of the maximum (“model.lb(strcmp(model.rxns,'BIOMASS_BS_10'))=0.95*growth_rate”). This prevents the model from sacrificing its growth rate to produce more byproducts, and therefore only determining what the highest effluxes are close to the biologically relevant growth rate. Then, the objective of the model was changed to be the exchange reaction corresponding to the metabolite of interest (e.g., “model = changeObjective(model, ‘EX_ac_e’)”), and the model was re-optimized. To identify the minimum flux, the model was re-optimized to minimize the flux [e.g., FBA = optimizeCbModel(model,'min')].

### Reaction essentiality

Reaction essentiality was performed in the COBRA Toolbox with the command relGrowth = singleRxnDeletion(model). This removes each reaction from the model one at a time and reoptimize it. Then, it returns the vector relGrowth, which is a series of numbers between 0 and 1, indicating the ratio of the growth rate with the corresponding reaction deleted to the “wild-type” growth rate with no deletions. These values for each strain were then recorded in a matrix of relative growth rates for each strain and reaction for further analysis. The cutoff for essentiality was a relative growth rate below 0.01.

### Pathway enumeration

To perform the pathway analysis, we first had to do flux sampling. Because the flux sampling methods are faster and more stable in the Python implementation, this was performed in COBRApy, unlike the rest of the analysis. First, the strain models were assembled in matlab and saved. Then, each strain model was loaded into COBRApy and sampled with 2,000 samples (“S = cobra.sampling.sample(model,2000)”). This was done in both the minimal glucose medium and with all exchanges open. The saved samples were then loaded into MATLAB, and the rank was calculated. Experimentation showed that the rank saturated after about 500 samples, as this was the dimensionality of the solution space. Additionally, 2,000 was chosen to be entirely certain all possibilities were represented.

### Computation

Most model simulations were performed in MATLAB R2022b using the COBRA Toolbox (70) version 3.4 and Gurobi 9.5.2, whereas flux sampling was performed in Python 3.10.12 COBRApy 0.27.0 (71), all in a UBUNTU 22.04.1 workstation.

## Data Availability

The pan-genome model, the files needed to reconstruct the strain specific models, and example code for doing so are available at https://github.com/mneal98/Bacillus_Pan_Genome_Model.

## SUPPLEMENTAL MATERIAL

Supplemental File Legends msystems.00923-24-s0001.docx Legends for the supplemental tables.

Table S1 msystems.00923-24-s0002.xlsx The list of genomes used in the study.

Table S2 msystems.00923-24-s0003.csv A binary matrix indicating which strains have which genes.

Table S3 msystems.00923-24-s0004.csv The second half of the binary matrix in Table S2.

Table S4 msystems.00923-24-s0005.xlsx Reaction identifiers, formulas, and other metadata to reconstruct the pan-genome model and binary matrix indicating which strains have which reactions.

Table S5 msystems.00923-24-s0006.xlsx Metabolite identifiers, descriptive names, and formulas from the pan-genome model.

Table S6 msystems.00923-24-s0007.xlsx The original Biolog plate readings for each strain, time point, and replicate.

Table S7 msystems.00923-24-s0008.xlsx Each strain in the model and which group it was assigned to.

Table S8 msystems.00923-24-s0009.xlsx The relative growth rates of each strain with each reaction deleted.

Table S9 msystems.00923-24-s0010.xlsx Identifiers and sequences for the representative annotated proteins that make up the pan-genome.

ASM does not own the copyrights to Supplemental Material that may be linked to, or accessed through, an article. The authors have granted ASM a non-exclusive, world-wide license to publish the Supplemental Material files. Please contact the corresponding author directly for reuse.

## References

[1] Errington J, Aart L. 2020. Microbe profile: Bacillus subtilis: model organism for cellular development, and industrial workhorse: this article is part of the microbe profiles collection. Microbiol (Reading) 166:425–427. doi:10.1099/mic.0.000922 PMC7376258 32391747

[2] Kunst F, Ogasawara N, Moszer I, Albertini AM, Alloni G, Azevedo V, Bertero MG, Bessières P, Bolotin A, Borchert S, et al.. 1997. The complete genome sequence of the Gram-positive bacterium Bacillus subtilis. Nature 390:249–256. doi:10.1038/36786 9384377

[3] Su Y, Liu C, Fang H, Zhang D. 2020. Bacillus subtilis: a universal cell factory for industry, agriculture, biomaterials and medicine. Microb Cell Fact 19:173. doi:10.1186/s12934-020-01436-8 32883293 PMC7650271

[4] Chicco D, Jurman G. 2020. The advantages of the Matthews correlation coefficient (MCC) over F1 score and accuracy in binary classification evaluation. BMC Genomics 21:6. doi:10.1186/s12864-019-6413-7 31898477 PMC6941312

[5] Schallmey M, Singh A, Ward OP. 2004. Developments in the use of Bacillus species for industrial production. Can J Microbiol 50:1–17. doi:10.1139/w03-076 15052317

[6] Brutscher LM, Borgmeier C, Garvey SM, Spears JL. 2022. Preclinical safety assessment of Bacillus subtilis BS50 for probiotic and food applications. Microorganisms 10:1038. doi:10.3390/microorganisms10051038 35630480 PMC9144164

[7] Bampidis V, Azimonti G, Bastos M de L, Christensen H, Dusemund B, Fašmon Durjava M, Kouba M, López‐Alonso M, López Puente S, Marcon F, Mayo B, Pechová A, Petkova M, Ramos F, Sanz Y, Villa RE, Woutersen R, Brozzi R, Galobart J, Pettenati E, Revez J, Innocenti ML, EFSA Panel on Additives and Products or Substances used in Animal Feed (FEEDAP). 2021. Safety and efficacy of an additive consisting of Bacillus subtilis DSM 32324 for all animal species (Chr. Hansen A/S). EFS2 19:e06523. doi:10.2903/j.efsa.2021.6523

[8] Tompkins TA, Xu X, Ahmarani J. 2010. A comprehensive review of post-market clinical studies performed in adults with an Asian probiotic formulation. Benef Microbes 1:93–106. doi:10.3920/BM2008.1005 21840798

[9] Kruse S, Pierre F, Morlock GE. 2021. Effects of the probiotic activity of Bacillus subtilis DSM 29784 in cultures and feeding stuff. J Agric Food Chem 69:11272–11281. doi:10.1021/acs.jafc.1c04811 34546731

[10] Stein T. 2005. Bacillus subtilis antibiotics: structures, syntheses and specific functions. Mol Microbiol 56:845–857. doi:10.1111/j.1365-2958.2005.04587.x 15853875

[11] Bais HP, Fall R, Vivanco JM. 2004. Biocontrol of Bacillus subtilis against infection of Arabidopsis roots by Pseudomonas syringae is facilitated by biofilm formation and surfactin production. Plant Physiol 134:307–319. doi:10.1104/pp.103.028712 14684838 PMC316310

[12] Yi Y, Luan P, Liu S, Shan Y, Hou Z, Zhao S, Jia S, Li R. 2022. Efficacy of Bacillus subtilis XZ18-3 as a biocontrol agent against Rhizoctonia cerealis on wheat. Agriculture 12:258. doi:10.3390/agriculture12020258

[13] Zhou Y, Li Q, Peng Z, Zhang J, Li J. 2022. Biocontrol effect of Bacillus subtilis YPS-32 on potato common scab and its complete genome sequence analysis. J Agric Food Chem 70:5339–5348. doi:10.1021/acs.jafc.2c00274 35467346

[14] Ku Y, Yang N, Pu P, Mei X, Cao L, Yang X, Cao C. 2021. Biocontrol mechanism of Bacillus subtilis C3 against bulb rot disease in Fritillaria taipaiensis P.Y.Li. Front Microbiol 12:756329. doi:10.3389/fmicb.2021.756329 34659191 PMC8515143

[15] Bahaddad SA, Almalki MHK, Alghamdi OA, Sohrab SS, Yasir M, Azhar EI, Chouayekh H. 2023. Bacillus species as direct-fed microbial antibiotic alternatives for monogastric production. Probiotics Antimicrob Proteins 15:1–16. doi:10.1007/s12602-022-09909-5 35092567 PMC8799964

[16] Gottel NR, Hill MS, Neal MJ, Allard SM, Zengler K, Gilbert JA. 2024. Biocontrol in built environments to reduce pathogen exposure and infection risk. ISME J 18:wrad024. doi:10.1093/ismejo/wrad024 38365248 PMC10848226

[17] Thiele I, Palsson BØ. 2010. A protocol for generating a high-quality genome-scale metabolic reconstruction. Nat Protoc 5:93–121. doi:10.1038/nprot.2009.203 20057383 PMC3125167

[18] Passi A, Tibocha-Bonilla JD, Kumar M, Tec-Campos D, Zengler K, Zuniga C. 2021. Genome-scale metabolic modeling enables in-depth understanding of big data. Metabolites 12:14. doi:10.3390/metabo12010014 35050136 PMC8778254

[19] Oh Y-K, Palsson BO, Park SM, Schilling CH, Mahadevan R. 2007. Genome-scale reconstruction of metabolic network in Bacillus subtilis based on high-throughput phenotyping and gene essentiality data. J Biol Chem 282:28791–28799. doi:10.1074/jbc.M703759200 17573341

[20] Henry CS, Zinner JF, Cohoon MP, Stevens RL. 2009. iBsu1103: a new genome-scale metabolic model of Bacillus subtilis based on SEED annotations. Genome Biol 10:R69. doi:10.1186/gb-2009-10-6-r69 19555510 PMC2718503

[21] Bi X, Cheng Y, Xu X, Lv X, Liu Y, Li J, Du G, Chen J, Ledesma-Amaro R, Liu L. 2023. etiBsu1209: a comprehensive multiscale metabolic model for Bacillus subtilis. Biotechnol Bioeng 120:1623–1639. doi:10.1002/bit.28355 36788025

[22] Barge NS, Sahoo A, Dasu VV. 2023. Analysis of the genome-scale metabolic model of Bacillus subtilis to design novel in-silico strategies for native and recombinant L-asparaginase overproduction. bioRxiv. doi:10.1101/2022.12.29.522229

[23] Liu Y, Zhang Q, Qi X, Gao H, Wang M, Guan H, Yu B. 2023. Metabolic engineering of Bacillus subtilis for riboflavin production: a review. Microorganisms 11:164. doi:10.3390/microorganisms11010164 36677456 PMC9863419

[24] Earl AM, Losick R, Kolter R. 2007. Bacillus subtilis genome diversity. J Bacteriol 189:1163–1170. doi:10.1128/JB.01343-06 17114265 PMC1797320

[25] Kiesewalter HT, Lozano-Andrade CN, Wibowo M, Strube ML, Maróti G, Snyder D, Jørgensen TS, Larsen TO, Cooper VS, Weber T, Kovács ÁT. 2021. Genomic and chemical diversity of Bacillus subtilis secondary metabolites against plant pathogenic fungi. mSystems 6:e00770-20. doi:10.1128/mSystems.00770-20 PMC8573961 33622852

[26] Kai M. 2020. Diversity and distribution of volatile secondary metabolites throughout Bacillus subtilis isolates. Front Microbiol 11:559. doi:10.3389/fmicb.2020.00559 32322244 PMC7156558

[27] Monk JM, Charusanti P, Aziz RK, Lerman JA, Premyodhin N, Orth JD, Feist AM, Palsson BØ. 2013. Genome-scale metabolic reconstructions of multiple Escherichia coli strains highlight strain-specific adaptations to nutritional environments. Proc Natl Acad Sci U S A 110:20338–20343. doi:10.1073/pnas.1307797110 24277855 PMC3864276

[28] Seif Y, Kavvas E, Lachance J-C, Yurkovich JT, Nuccio S-P, Fang X, Catoiu E, Raffatellu M, Palsson BO, Monk JM. 2018. Genome-scale metabolic reconstructions of multiple Salmonella strains reveal serovar-specific metabolic traits. Nat Commun 9:3771. doi:10.1038/s41467-018-06112-5 30218022 PMC6138749

[29] Bosi E, Monk JM, Aziz RK, Fondi M, Nizet V, Palsson BØ. 2016. Comparative genome-scale modelling of Staphylococcus aureus strains identifies strain-specific metabolic capabilities linked to pathogenicity. Proc Natl Acad Sci U S A 113:E3801–E3809. doi:10.1073/pnas.1523199113 27286824 PMC4932939

[30] Correia K, Mahadevan R. 2020. Pan-genome-scale network reconstruction: harnessing phylogenomics increases the quantity and quality of metabolic models. Biotechnol J 15:1900519. doi:10.1002/biot.201900519 32744423

[31] Norsigian CJ, Fang X, Seif Y, Monk JM, Palsson BO. 2020. A workflow for generating multi-strain genome-scale metabolic models of prokaryotes. Nat Protoc 15:1–14. doi:10.1038/s41596-019-0254-3 31863076 PMC7017905

[32] Tantoso E, Eisenhaber B, Kirsch M, Shitov V, Zhao Z, Eisenhaber F. 2022. To kill or to be killed: pangenome analysis of Escherichia coli strains reveals a tailocin specific for pandemic ST131. BMC Biol 20:146. doi:10.1186/s12915-022-01347-7 35710371 PMC9205054

[33] Page AJ, Cummins CA, Hunt M, Wong VK, Reuter S, Holden MTG, Fookes M, Falush D, Keane JA, Parkhill J. 2015. Roary: rapid large-scale prokaryote pan genome analysis. Bioinformatics 31:3691–3693. doi:10.1093/bioinformatics/btv421 26198102 PMC4817141

[34] Tännler S, Decasper S, Sauer U. 2008. Maintenance metabolism and carbon fluxes in Bacillus species. Microb Cell Fact 7:19. doi:10.1186/1475-2859-7-19 18564406 PMC2442585

[35] Bochner BR, Gadzinski P, Panomitros E. 2001. Phenotype microarrays for high-throughput phenotypic testing and assay of gene function. Genome Res 11:1246–1255. doi:10.1101/gr.186501 11435407 PMC311101

[36] Dauner M, Sauer U. 2001. Stoichiometric growth model for riboflavin-producing Bacillus subtilis. Biotechnol Bioeng 76:132–143. doi:10.1002/bit.1153 11505383

[37] Meyer FM, Stülke J. 2013. Malate metabolism in Bacillus subtilis: distinct roles for three classes of malate-oxidizing enzymes. FEMS Microbiol Lett 339:17–22. doi:10.1111/1574-6968.12041 23136871

[38] Fisher SH. 1999. Regulation of nitrogen metabolism in Bacillus subtilis: vive la différence! Mol Microbiol 32:223–232. doi:10.1046/j.1365-2958.1999.01333.x 10231480

[39] Nygaard P, Duckert P, Saxild HH. 1996. Role of adenine deaminase in purine salvage and nitrogen metabolism and characterization of the ade gene in Bacillus subtilis. J Bacteriol 178:846–853. doi:10.1128/jb.178.3.846-853.1996 8550522 PMC177734

[40] Rima BK, Takahashi I. 1977. Metabolism of pyrimidine bases and nucleosides in Bacillus subtilis. J Bacteriol 129:574–579. doi:10.1128/jb.129.2.574-579.1977 402352 PMC234978

[41] He H, Li Y, Zhang L, Ding Z, Shi G. 2023. Understanding and application of Bacillus nitrogen regulation: a synthetic biology perspective. J Adv Res 49:1–14. doi:10.1016/j.jare.2022.09.003 36103961 PMC10334148

[42] Patel D, Buch A. 2019. Aerobic L-tartrate utilization by Bacillus isolates. J Pure Appl Microbiol 13:2045–2054. doi:10.22207/JPAM.13.4.16

[43] Duport C, Madeira J-P, Farjad M, Alpha-Bazin B, Armengaud J. 2021. Methionine sulfoxide reductases contribute to anaerobic fermentative metabolism in Bacillus cereus. Antioxidants (Basel) 10:819. doi:10.3390/antiox10050819 34065610 PMC8161402

[44] Gudmundsson S, Thiele I. 2010. Computationally efficient flux variability analysis. BMC Bioinformatics 11:489. doi:10.1186/1471-2105-11-489 20920235 PMC2963619

[45] Reed JL, Palsson BØ. 2004. Genome-scale in silico models of E. coli have multiple equivalent phenotypic states: assessment of correlated reaction subsets that comprise network states. Genome Res 14:1797–1805. doi:10.1101/gr.2546004 15342562 PMC515326

[46] Bernstein DB, Sulheim S, Almaas E, Segrè D. 2021. Addressing uncertainty in genome-scale metabolic model reconstruction and analysis. Genome Biol 22:64. doi:10.1186/s13059-021-02289-z 33602294 PMC7890832

[47] Rao MRR, Stokes JL. 1953. Utilization of ethanol by acetic acid bacteria. J Bacteriol 66:634–638. doi:10.1128/jb.66.6.634-638.1953 13117785 PMC317453

[48] Kurkivuori J, Salaspuro V, Kaihovaara P, Kari K, Rautemaa R, Grönroos L, Meurman JH, Salaspuro M. 2007. Acetaldehyde production from ethanol by oral streptococci. Oral Oncol 43:181–186. doi:10.1016/j.oraloncology.2006.02.005 16859955

[49] Roine RP, Salmela KS, Salaspuro M. 1995. Alcohol metabolism in Helicobacter pylori-infected stomach. Ann Med 27:583–588. doi:10.3109/07853899509002473 8541036

[50] Bordbar A, Nagarajan H, Lewis NE, Latif H, Ebrahim A, Federowicz S, Schellenberger J, Palsson BO. 2014. Minimal metabolic pathway structure is consistent with associated biomolecular interactions. Mol Syst Biol 10:737. doi:10.15252/msb.20145243 24987116 PMC4299494

[51] Fu Y, Liu X, Su Z, Wang P, Guo Q, Ma P. 2023. Arabinose plays an important role in regulating the growth and sporulation of Bacillus subtilis NCD-2. Int J Mol Sci 24:17472. doi:10.3390/ijms242417472 38139303 PMC10744016

[52] Sekowska A, Mulard L, Krogh S, Tse JK, Danchin A. 2001. MtnK, methylthioribose kinase, is a starvation-induced protein in Bacillus subtilis. BMC Microbiol 1:15. doi:10.1186/1471-2180-1-15 11545674 PMC55331

[53] Heilbronn J, Wilson J, Berger BJ. 1999. Tyrosine aminotransferase catalyzes the final step of methionine recycling in Klebsiella pneumoniae. J Bacteriol 181:1739–1747. doi:10.1128/JB.181.6.1739-1747.1999 10074065 PMC93571

[54] Duan YX, Chen T, Chen X, Zhao XM. 2010. Overexpression of glucose-6-phosphate dehydrogenase enhances riboflavin production in Bacillus subtilis. Appl Microbiol Biotechnol 85:1907–1914. doi:10.1007/s00253-009-2247-6 19779711

[55] Klein W, Weber MHW, Marahiel MA. 1999. Cold shock response of Bacillus subtilis: isoleucine-dependent switch in the fatty acid branching pattern for membrane adaptation to low temperatures. J Bacteriol 181:5341–5349. doi:10.1128/JB.181.17.5341-5349.1999 10464205 PMC94040

[56] Bell TH, Bell T. 2020. Many roads to bacterial generalism. FEMS Microbiol Ecol 97:fiaa240. doi:10.1093/femsec/fiaa240 33238305

[57] Hu G, Wang Y, Blake C, Nordgaard M, Liu X, Wang B, Kovács ÁT. 2023. Parallel genetic adaptation of Bacillus subtilis to different plant species. Microb Genom 9:mgen001064. doi:10.1099/mgen.0.001064 37466402 PMC10438812

[58] Nordgaard M, Blake C, Maróti G, Hu G, Wang Y, Strube ML, Kovács ÁT. 2022. Experimental evolution of Bacillus subtilis on Arabidopsis thaliana roots reveals fast adaptation and improved root colonization. iScience 25:104406. doi:10.1016/j.isci.2022.104406 35663012 PMC9157203

[59] Mutlu A, Kaspar C, Becker N, Bischofs IB. 2020. A spore quality–quantity tradeoff favors diverse sporulation strategies in Bacillus subtilis. ISME J 14:2703–2714. doi:10.1038/s41396-020-0721-4 32724142 PMC7784978

[60] Nuhamunada M, Mohite OS, Phaneuf PV, Palsson BO, Weber T. 2024. BGCFlow: systematic pangenome workflow for the analysis of biosynthetic gene clusters across large genomic datasets. Nucleic Acids Res 52:5478–5495. doi:10.1093/nar/gkae314 38686794 PMC11162802

[61] Parks DH, Imelfort M, Skennerton CT, Hugenholtz P, Tyson GW. 2015. CheckM: assessing the quality of microbial genomes recovered from isolates, single cells, and metagenomes. Genome Res 25:1043–1055. doi:10.1101/gr.186072.114 25977477 PMC4484387

[62] Seemann T. 2014. Prokka: rapid prokaryotic genome annotation. Bioinformatics 30:2068–2069. doi:10.1093/bioinformatics/btu153 24642063

[63] Monk JM, Lloyd CJ, Brunk E, Mih N, Sastry A, King Z, Takeuchi R, Nomura W, Zhang Z, Mori H, Feist AM, Palsson BO. 2017. iML1515, a knowledgebase that computes Escherichia coli traits. Nat Biotechnol 35:904–908. doi:10.1038/nbt.3956 29020004 PMC6521705

[64] Becker SA, Palsson BØ. 2005. Genome-scale reconstruction of the metabolic network in Staphylococcus aureus N315: an initial draft to the two-dimensional annotation. BMC Microbiol 5:8. doi:10.1186/1471-2180-5-8 15752426 PMC1079855

[65] Seif Y, Monk JM, Mih N, Tsunemoto H, Poudel S, Zuniga C, Broddrick J, Zengler K, Palsson BO. 2019. A computational knowledge-base elucidates the response of Staphylococcus aureus to different media types. PLOS Comput Biol 15:e1006644. doi:10.1371/journal.pcbi.1006644 30625152 PMC6326480

[66] Flahaut NAL, Wiersma A, van de Bunt B, Martens DE, Schaap PJ, Sijtsma L, Dos Santos VAM, de Vos WM. 2013. Genome-scale metabolic model for Lactococcus lactis MG1363 and its application to the analysis of flavor formation. Appl Microbiol Biotechnol 97:8729–8739. doi:10.1007/s00253-013-5140-2 23974365

[67] King ZA, Lu J, Dräger A, Miller P, Federowicz S, Lerman JA, Ebrahim A, Palsson BO, Lewis NE. 2016. BiGG Models: a platform for integrating, standardizing and sharing genome-scale models. Nucleic Acids Res 44:D515–D522. doi:10.1093/nar/gkv1049 26476456 PMC4702785

[68] TCDB. HOME. Available from: https://tcdb.org. Retrieved 24 Mar 2022.

[69] Mackie AM, Hassan KA, Paulsen IT, Tetu SG. 2014. Biolog Phenotype MicroArrays for phenotypic characterization of microbial cells, p 123–130. In Paulsen IT, Holmes AJ (ed), Environmental microbiology. Humana Press, Totowa, NJ. 10.1007/978-1-62703-712-9_10 24515365

[70] Heirendt L, Arreckx S, Pfau T, Mendoza SN, Richelle A, Heinken A, Haraldsdóttir HS, Wachowiak J, Keating SM, Vlasov V, et al.. 2019. Creation and analysis of biochemical constraint-based models using the COBRA Toolbox v.3.0. Nat Protoc 14:639–702. doi:10.1038/s41596-018-0098-2 30787451 PMC6635304

[71] Ebrahim A, Lerman JA, Palsson BO, Hyduke DR. 2013. COBRApy: constraints-based reconstruction and analysis for python. BMC Syst Biol 7:74. doi:10.1186/1752-0509-7-74 23927696 PMC3751080

