# Supplementary material for: Pan-genome-scale metabolic modeling of Bacillus subtilis reveals functionally distinct groups
Source: mSystems. 2024 Oct 4;9(11):e00923-24. doi: 10.1128/msystems.00923-24 (PMC11575223; doi:10.1128/msystems.00923-24)
Supplement: Supplemental File Legends — Legends for the supplemental tables. [file msystems.00923-24-s0001.docx]

Table S1: The list of genomes used in the study, with BioProject, BioSample, and GenBank identifiers.

Table S2: A binary matrix indicating which strains have which genes, as a .csv file. A "1" indicates that the gene labeled along the row is present in the strain indicated by the column identifier. This is the first half of the matrix; the second half is in Table S3.

Table S3: The second half of the binary matrix in Table S2.

Table S4: Sheet 1 contains the reaction identifiers, formulas, and other metadata to reconstruct the pan-genome model. The exchange reaction lower bounds are set to the minimal glucose medium describes in the manuscript. Sheet 2 contains a binary matrix indicating which strains have which reactions. A "1" indicates that the strain given by the column identifier satisfies the genetic requirements to perform the reaction given by the row identifier.

Table S5: Metabolite identifiers, descriptive names, and formulas used in the pan-genome model.

Table S6: The original Biolog plate readings for each strain, time point, and replicate. Each row corresponds to one flattened plate reading, i.e. the absorbance values for all wells for a given strain, time, and replicate.

Table S7: Each strain in the model paired to a number indicating which of the five groups it was assigned to.

Table S8: The relative growth rates of each strain with each reaction deleted. A "1" indicates that deleting this reaction had no effect on the growth of the organism, either because it was entirely unessential or because the strain did not have that reaction to begin with. A numerical value less than one indicates the proportion of growth that remained, e.g. "0.5" indicates that the strain grew at only half the rate it did previously when that reaction was knocked out.

Table S9: Identifiers and sequences for the representative annotated proteins that make up the pan-genome. These were the sequences used to BLAST against template models to create the draft reconstruction.
